# Causal Modelling for Supporting Planning and Management of Mental Health Services and Systems: A Systematic Review

**DOI:** 10.3390/ijerph16030332

**Published:** 2019-01-25

**Authors:** Nerea Almeda, Carlos R. García-Alonso, José A. Salinas-Pérez, Mencía R. Gutiérrez-Colosía, Luis Salvador-Carulla

**Affiliations:** 1 Universidad Loyola Andalucía, Department of Psychology, C/Energía Solar 1, 41014 Seville, Spain; menciaruiz@uloyola.es; 2 Universidad Loyola Andalucía, Department of Quantitative Methods, C/Energía Solar 1, 41014 Seville, Spain; cgarcia@uloyola.es (C.G.-A.); jsalinas@uloyola.es (J.A.S.-P.); 3 Centre for Mental Health Research, Research School of Population Health, Australian National University, 63 Eggleston Rd, Acton, ACT 2601, Australia; luis.salvador-carulla@anu.edu.au

**Keywords:** mental health systems, mental health services, mental health care, management, policy-making, planning, causal model, Bayesian networks, structural equation modelling, systematic review

## Abstract

Mental health services and systems (MHSS) are characterized by their complexity. Causal modelling is a tool for decision-making based on identifying critical variables and their causal relationships. In the last two decades, great efforts have been made to provide integrated and balanced mental health care, but there is no a clear systematization of causal links among MHSS variables. This study aims to review the empirical background of causal modelling applications (Bayesian networks and structural equation modelling) for MHSS management. The study followed the PRISMA guidelines (PROSPERO: CRD42018102518). The quality of the studies was assessed by using a new checklist based on MHSS structure, target population, resources, outcomes, and methodology. Seven out of 1847 studies fulfilled the inclusion criteria. After the review, the selected papers showed very different objectives and subjects of study. This finding seems to indicate that causal modelling has potential to be relevant for decision-making. The main findings provided information about the complexity of the analyzed systems, distinguishing whether they analyzed a single MHSS or a group of MHSSs. The discriminative power of the checklist for quality assessment was evaluated, with positive results. This review identified relevant strategies for policy-making. Causal modelling can be used for better understanding the MHSS behavior, identifying service performance factors, and improving evidence-informed policy-making.

## 1. Introduction

Throughout the history of psychiatric care, the deinstitutionalization process has constituted an inflexion point in mental health care provision. With the decline of asylums in the mid-fifties and their closure in the eighties in Europe and the United States of America, mental health care delivery was shifted from isolated hospitals and asylums to communities [1,2]. Community mental health care is understood as the promotion of mental health for a target population, considering its needs and strengths, favouring social support, and highlighting evidence-based and recovery-oriented services [3]. Nevertheless, the provision of integrated care into the community is still a major challenge [4] due to the added complexity of mental health systems [5,6].

The current mental health environment is characterized by high levels of mental disorders [7], socioeconomic costs [8,9,10], and unmet population needs [11,12,13,14], which require improved planning and management of mental health services and systems (MHSS). Trying to use an approach from an ecological perspective, four levels of analysis can be defined: macro, meso, micro, and nano levels. This framework is useful for classifying models in order to know the levels at which they can have a potential impact. The majority of studies focus on analyzing the nano level, which involves patient or consumer interventions [15,16,17,18] and career and professional characteristics [19,20]. The other levels have been less frequently studied, although they are relevant for planning and management of mental health care. Thus, micro-level analyses include organizations for care provision (for example, mental health centres or acute wards), meso-level analyses include local information (for example, small mental health catchment areas), and macro-level analyses comprise global information (large health districts, regional or national) [21,22]. Findings from environmental sciences show that advanced methodologies, modelling, and real simulations can play an important role in guiding evidence-informed policy design based on the analysis of healthcare ecosystems for providing better mental health care [6]. Recent studies focused on developing decision support systems (DSS) show that expert knowledge formalization is fundamental to guide both operational and statistical techniques [23,24]. A DSS is a computer-based tool that usually integrates databases with analytical procedures [24] (operational like relative efficiency analysis, statistical like factor analysis, artificial intelligence like fuzzy inference, etc.). These tools are designed and developed for processing data and producing useful information for decision makers in complex and uncertain environments. In some cases, resulting formal representations of the explicit knowledge (rule-based models, causal models, etc., obtained from a knowledge discovery process) have been integrated into analytical procedures (operational, statistical, etc.) [5]. A knowledge discovery process includes qualitative (focus and Delphi groups, structured interviews, etc.) and quantitative techniques (knowledge discovery from data, cluster analysis, factor analysis, etc.) in order to make expert knowledge explicit. The final product of this process is a structured model (hierarchical, causal, rule-based, etc.) that can be included in, for example, DSS [5,24]. One of the best ways to do so is to design a causal model wherein all the critical variables and dimensions are identified, as well as their causal relationships [25], if they exist.

A causal model, or its formal expression, a Bayesian network, shows causes and effects [26,27] by using variables (nodes in, usually, a graph) and their relationships (connections—different kinds of arrows or lines—in, again, graphs). Bayesian networks (BNs) can be integrated in DSS for explaining causal links between variables for both operational and statistical analysis because in real systems, variables and/or dimensions (that summarize the behaviour of a set of variables with a specific meaning, for example, deprivation) cannot be considered exogenous (there are causes and effects). Very often, BNs are theoretical models, but if data are available, their structure (variables/dimensions and causal links) can be tested and, sometimes, confirmed by using statistical procedures, such as structural equations modelling (SEM) [25]. 

The formal structure of a causal model can be defined as a set of equations (usually represented by a graph), such as:

(1) $$x_{i}=f_{i}\left(pr_{i},u_{i}\right),\;i=1,2,\ldots ,n$$

where $x_{i}$ is the value of the *i*th variable (*n* is the number of variables of the model); $pr_{i}$ is the minimum set of predecessor variables of $x_{i},$ which are Markovian parents that make $x_{i}$ independent of all its other predecessors; and, finally, $u_{i}$ is the error that assumes the existence of unobserved variables, factors, or relationships. The Markovian parents are the minimum set of variables that can be considered direct causes of $x_{i}$ and, therefore, they have a direct and unidirectional link to $x_{i}$ in a BN. This model (1) is perfectly causal, nonparametric, and nonlinear and is therefore more general than the structural equations that, in linear form, are defined by a set of equations, such as:

(2) $$x_{i}=\sum_{j=1}^{m}\beta_{ij}x_{j}+u_{i},\;i=1,2,\ldots ,n$$

where $x_{j}$ represents the independent variables (variables than can be considered causes of $x_{i}$), and $\beta_{ij}$ represents the structural coefficients of $x_{j}$. The structural equations model (2)—always algebraic – can be more general if it includes nonlinear equations, but it can be difficult to solve. In this situation, the problem is answering the following question: can we consider the structural coefficients calculated using a mathematical procedure as a representation of causal behaviour? This question is fundamental and not easy to answer [25] because causal relationships cannot be derived from any statistical or functional operation. Causal relationships derive from previous causal assumptions that have to be formalized in an appropriate way like, for example, graphs [25,27]. By using graphs as a mathematical language, researchers can overcome one of the main drawbacks in (2), that is: causal relationships between two variables cannot be correctly defined without taking into account that the cause can also have cause variables [25]. Therefore, the effect of the latter cannot be separated when the effect variable is assessed. Taking into account this circumstance, we have added structural equations in the search strategy in order to check the influence of causal reasoning in the resulting studies.

In health care, BNs have been applied for decision-making [28,29,30,31] and case assessment: analyzing new diagnosis strategies [32,33,34] and diagnosing social anxiety [35], depression [36,37], and Alzheimer’s disease [38,39]. Despite its reported utility in formalising the explicit knowledge about the structure of a system, in assessing potential responses:

(3) $$X_{M_{x_{j}}}^{i}\left(u\right)$$

where $X^{i}$ and $X_{j}$ are two subsets in the set of variables –endogenous or exogenous (U)- of the BN and $M_{x_{j}}$ is the action:

(4) $$do\left(X_{j}=x_{j},\forall j\right)$$

on it (system) and in evaluating counterfactual sentences (in situation u, $X^{i}$ would be $x_{i}$, had $X_{j}$ been $x_{j}$) (3,4), the application of BNs for the analysis of health care services and systems is scarce. A number of studies have used BNs for assessing the quality of nursing homes [40], but it is not a frequent method for planning or management. In mental health, the design of a BN is complex because all the organizations involved must be coordinated at the micro, meso, and macro levels [22]. Although there is an abundant background concerning the provision of mental health care in the community [41,42,43,44,45,46,47], there is still no clear identification of its critical variables (inputs and outcomes of the system) and their causal links (causes and effects of any real intervention). For example, the number of persons in staff is a consequence of the number of places, beds, or programs or vice versa. 

The aim of this paper is to systematically review the empirical background of causal modelling applications by employing a BN and SEM for planning and management of MHSS. Both the studies and the most relevant strategies for policy-making are identified.

## 2. Materials and Methods 

We followed the Preferred Reporting Items for Systematic Reviews and Meta-Analyses (PRISMA) guidelines [48] for systematically reviewing the literature. This study has been registered in the International Prospective Register of Systematic Reviews database (PROSPERO number CRD42018102518). In order to facilitate the reading of this study, we have specified the acronyms used in Table 1.

### 2.1. Search Strategy

Several search strategies were explored. First, we aimed to identify potential studies that applied BNs for supporting decision-making in mental health. We designed an inclusive Boolean algorithm including specific terms for psychopathology and BNs (Table 2). After carrying out the procedure, 20 studies focused on BNs and mental health care, with the majority focusing on case assessment and diagnosis of psychopathologies (nano level). In addition, it was detected that SEM was frequently used without mentioning its relationship (see the introduction) with causal models. Pearl [25] stated there exists a potential risk of using SEM, like, for example, a regression model that can show spurious causal links. These kind of analyses are not based on a real BN developed previously by using explicit expert knowledge for explaining the causal nature of the system under study. In BN, causal links represent real cause-effect relationships between variables (domains and/or constructs can also be included) that sometimes, if complete and reliable datasets are available, can be confirmed by using statistical analysis like SEM. 

Consequently, a second and definitive search strategy was designed to solve all the limitations mentioned above, including new terms related to mental health services (to avoid terminological problems in mental health services classifications), SEM, and planning and management. This search strategy was piloted on April 1, 2018. The final Boolean algorithm was conducted in the MEDLINE database (PubMed version) (Table 3). At a third stage, we conducted the search in the following databases: Scopus, Web of Science, PsycARTICLES, PsycINFO, Psychology Database, Nursing & Allied Health Database, and Health & Medical Collections. 

The search strategy was developed based on the PICO Model:

1. Population (P): All types of mental health services and/or systems that provide care for the population with a lived experience of mental disorder. Due to the wide terminological variability relating to MHSS, an inclusive set of terms was included [49,50,51,52,53] (Table 3).
2. Intervention (I): Causal modelling, including BNs and SEM (as a simplification of a BN), for guiding and supporting planning and management of MHSS [54].
3. Comparator (C): Refers to a control group or comparison intervention (PICO is a guide for designing research questions based on structured search strategies in a clinical framework). In our study, it is not applicable.
4. Outcome (O): Any resulting expert-based or data-based causal model.

### 2.2. Eligibility Criteria

We included studies that employed BNs and SEM for supporting planning and management of mental health services and/or systems. Only peer-reviewed articles and book chapters were selected (no constraints due to country of origin, publication date, or language were taken into consideration), and other publication types were excluded. 

### 2.3. Study Selection, Data Collection, and Summary Measures

After piloting the Boolean algorithm, we removed duplicated results of the record pool. CG and NA independently carried out the selection procedure in two phases: screening and eligibility. In the first step, NA and CG checked paper titles and abstracts to decide if they met the inclusion criteria. In the second step, they reviewed their full text. JAS, a third author not involved in the selection process, resolved any disagreement between CG and NA. The concordance degree was assessed by statistical tests (kappa and ICC).

Data extracted were organized into five sections: (1) study selection, (2) study characteristics, (3) main findings, (4) quality of included studies, and (5) implications for policy-making. In the “study selection” section, we describe the process for selecting studies (screening and eligibility). “Study characteristics” involves information regarding the country and year, objectives, type of MHSS, target population, data, variables, and methods. The “quality of included studies” section offers a new proposal for the assessment of the study quality. Finally, the “implications for policy-making in mental health care” section identifies potential strategies for management and planning.

A meta-analysis of the information available was not developed because of the extreme variability of the studies found (their findings are not comparable). 

### 2.4. Quality Assessment

To assess the quality of the studies included, a new checklist was designed based on MHSS structure [51,53,55], target population for care delivery, and causality [25,54]. In addition, we took into consideration a specific quality assessment tool developed by Thomas, Ciliska, Dobbins and Micucci [56] for evaluating systematic literature reviews assessing the effectiveness of public health nursing interventions. The items finally selected for quality assessment were: the study includes more than one type of mental health service or system (one item related to the MHSS structure under study); the study specifies more than one type of target population for care delivery (one item related to the characteristics of the target population); the study analyzes variables, including resources and outcomes of the mental health care (one item related to the existence of resources and outcomes); and the study includes a causal graph, takes into account external expert knowledge for identifying the nodes and the causal relationships of the causal graph, combines data and external expert-based knowledge, includes sensitivity or parametric analysis, carries out factorial confirmatory/exploratory analysis, develops any kind of causal-related inference and, finally, the causal model is integrated in a decision support system (seven items related to causal methodology). Due to the purpose of the systematic review, the weight of methodological issues is greater than items related to the search strategy (Table 3): types of mental health services and management and planning. To assess the relevance of causal modelling, three domains (groups of items that can be considered essential in this kind of studies) have been taken into consideration: (1) expert-based issues (existence of a causal graph, expert identification of the nodes, and knowledge inclusion), (2) statistical procedures (sensitivity/parametric/factor analysis and causal-related inference), and (3) managerial implications (decision support systems). These domains balance the relevance of the knowledge, permit statistical analysis, and introduce practical implications for management.

## 3. Results

### 3.1. Study Selection

**Figure 1:**
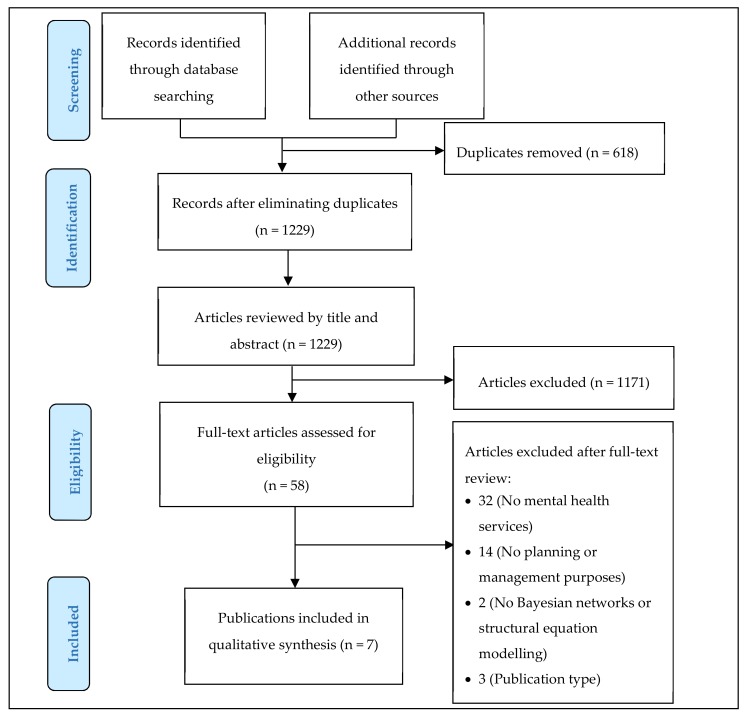
Flow chart of articles included and excluded after the systematic review.

In total, the search strategy retrieved 1847 records (Figure 1); the bibliography of the selected studies was also checked, and no additional references were found. After removing duplicates, 1229 records were analysed (CG and NA) by titles and abstracts: 58 fulfilled inclusion criteria. These 58 studies were thoroughly assessed (CG and NA) for eligibility (full text) and, finally, seven articles fulfilled the inclusion criteria (Figure 1). The main reasons for rejecting a study were (in order, more to less important): the object of study was a group of patients or specific illnesses and their methods were not really used to develop a BN or an SEM or were mainly used in diagnosis. The degree of agreement was assessed by using the intra-class correlation (ICC) analysis and Kappa index. As expected, the results evidenced that there was a strong agreement level (*Kappa* = 0.972, *p* = 0.000; *ICC* = 0.986, *p* = 0.000).

*From:* Moher D, Liberati A, Tetzlaff J, Altman DG, the PRISMA Group (2009). Preferred Reporting Items for Systematic Reviews and Meta-Analyses: The PRISMA Statement. PLoS Med 6(7): e1000097. doi:10.1371/journal.pmed1000097 

### 3.2. Study Characteristics

#### 3.2.1. Country and Year

High variations in publication dates and countries were found (Table 4). The first study was published forty years ago [57], while the last one was recently published [58]. Since the first publication, the production of studies has been irregular. Pioneer studies were from United States of America [57,59] and were published between the 1980s and 1990s; no other study was published until 2012 [60]. 

#### 3.2.2. Objectives

The selected studies show very different aims (Table 4). In [61], causal reasoning is used to develop a model for discharged decision-making in medium secure services. In the Delany, Fletcher and Lennox study [59], it is used for testing the impact of the structure of shelter organizations on services-amenities and organizational relations. For Roux, Passerieux and Fleury [58], the purpose was to assess the service performance as a mediating factor between patient needs and outcome production. Finally, two papers aimed to identify determinants of long-term care services use [57] and trends in hospital and community care over 14 years [62]. The remaining two studies wanted to identify evaluation criteria for mental health care quality assessment [60] and to improve relative technical efficiency assessment [22]. 

#### 3.2.3. Types of Mental Health Services and Systems

Studies can be divided into two main groups, depending on whether they analyzed a single MHSS or a group of MHSSs (Table 4). Four studies examined a single MHSS and the services analyzed were medium secure services [61]; sheltered homes [59]; mental hospitals [60]; and residential services, including mental hospitals, general hospitals, and nursing homes care [57]. On the other hand, three studies [22,58,62] combined hospital and community-based care services, following a systems approach and analysis. 

#### 3.2.4. Target Population

In all studies, the target population was people with a lived experience of mental disorder. Two studies [58,62] used an international diagnostic criteria, such as DSM-5 or ICD-10, for classifying psychiatric cases. Three studies focused on general mental health diagnosis, but they did not specify the diagnostic criteria employed [22,60,61]. Finally, two studies were focused on specific target groups: homeless [59] and/or elderly people with long-term care needs [57] (Table 4). 

#### 3.2.5. Data

Six out of seven studies collected data by using questionnaires or surveys [57,58,59,60,61,62] (Table 4). Some used data retrieved at the national level, for example, from the Police National Computer [61], NHS England [62], state-operated hospitals in Korea [60], Mental Health Service Network from Quebec [58], Public Mental Health System of Andalusia [22], and Massachusetts Department of Public Health [57]. In the study of Salvador-Carulla et al. [22], data were used for assessing the relative technical efficiency, not for checking the BN, which showed the causal relationships between variables. Again, many sources had different purposes.

#### 3.2.6. Variables

BNs [22,61] included greater numbers of variables than SEM [57,58,59,60,62] (Table 4). Due to the variability within the analyzed variable sets, three categories have been designed to classify them: resources, service user’s characteristics, and service performance and outcomes (Table 4). 

#### 3.2.7. Methods

Regarding the causal methodology, both BNs and SEM were identified in the selected studies. Five out of seven applied SEM and data-based causal models [57,58,59,60,62] combined with additional statistical methods (Table 4). 

On the other hand, two out of seven studies [22,61] developed a BN. Constantinou et al. [61] tested the causal predictability of their BN by carrying out a quantitative analysis. In Salvador-Carulla et al. [22], formalized expert knowledge (standard if … then … rule-base model) from senior managers and policy-makers was used for designing the BN and for interpreting variable values (Table 4).

BNs used more variables than SEM. In addition, BNs were more flexible because they could include almost any causal information, but SEM depends on the availability of data. 

**Figure 2:**
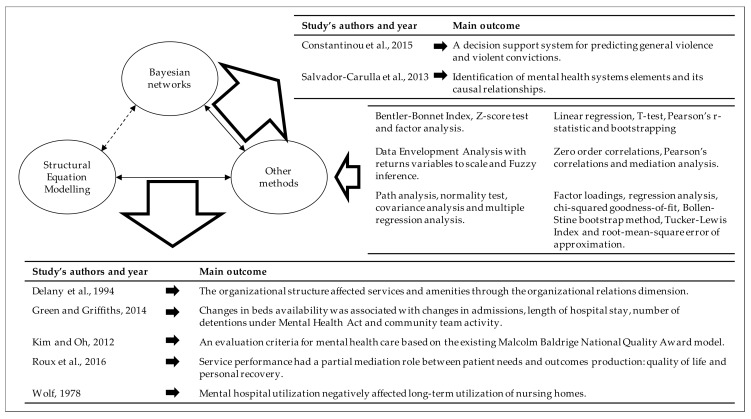
Formal representation of the selected studies (Note: no study combines Bayesian networks and Structural Equation Modelling).

The methods carried out in the seven studies and their main outcomes are represented in Figure 2. 

### 3.3. Main Findings

The main findings of the selected studies were classified according to the complexity of the systems under analysis, distinguishing if they focused their attention on a single MHSS or a group of MHSSs and including the levels (macro, meso, micro, and nano) at which the designed models can have a potential impact (Table 5). Therefore, complexity is directly related to the potential number of variables and relationships needed to explain the whole environment under study. Regarding the number of MHSSs, the DSVM-MSS model (micro level) is an appropriate DSS for predicting if a service user is ready to be discharged from medium-secure services [61], and this model introduces moderate to significant improvements in comparison with the methodology currently used (clinical or regression-based models). In shelter homes (micro level), the effect of the organizational structure on the service and amenities performance is better through organizational relations. Therefore, organizational relations represent a mediator between organizational structure and services and amenities [59]. The case of mental health hospitals (micro level), which analyzed the structure of the Malcolm Baldrige National Quality Award model, showed that Driver, Direction, System, and Foundation elements of the model had a variable impact among them and/or on the results [60]. In addition, sociocultural variables explained only 9% of the variance of mental hospital utilization, at the meso level [57].

Following with studies that included a group of MHSSs (Table 5), the results showed that the pattern of inpatient admissions and length of stay varied across different psychopathologies over 14 years, at the micro level (1998-2012) [62]. In addition, there was a significant association between patient needs, service performance, and/or outcomes produced at the micro level [58]. Finally, the EbCA-BNW-DEA model, at the meso level, was an appropriate decision-making tool for supporting planning and management of MHSSs, as it improved efficiency assessment, included expert knowledge, and established causal relationships among their elements [22].

Finally, all the studies that used SEM linked their results to causal structures. As it was stated before, this fact can be arguable. Looking for evidence in that sense, the references cited in the selected studies were studied. Only [22,61] developed a BN (Figure 2) and directly cited Judea Pearl´s research (Adnan Darwiche is not cited at all). Checking the references cited by the selected studies, only [61], 17 studies, and [58], one study, include Pearl´s research, and Darwiche´s research is not cited at all. On the other hand, no selected study has designed any counterfactual sentence related to their causal models.

### 3.4. Quality of Included Studies

The quality assessment of selected studies involved four components: MHSS structure, target population, resources and outcomes, and methodology (Table 6). 

Three out of seven studies [22,58,62] included more than one type of mental health service under analysis and the correspondent diverse target population (not restricted to a specific target population). All studies integrated variables of resources and outcomes of the MHSSs in the causal model.

Regarding the methodology, six out of the seven studies included a graph defining variables (nodes) and causal relationships (links) [22,57,58,59,60,61]. Four out of the seven studies included external expert knowledge for designing the graph [22,57,60,61]. Four studies combined data and external expert-based knowledge [57,58,60,61]. Following with the methods, four studies used sensitivity or parametric analysis [58,59,61,62], while the development of factorial confirmatory or exploratory analysis was included in three studies [57,59,60]. In addition, two studies included causal-related inference [61,62]. Finally, two studies integrated a BN in a more complex DSS [22,61].

Analyzing the checklist for quality assessment (Table 6), item 3 (variables include resources and outcomes of mental health care) was not discriminative and must be removed because all the studies matched with it.

According to the number of fulfilled items, the best study is [61] because it fulfils six (100% methodological items) out to nine items (after the exclusion of item 3); in second position are [22,58], with five (60% methodological) out nine items; in third are [57,60], with four items (100% methodological); in fourth is the study [62], which fulfils four items, only 20% of which are methodological ones; and finally, [59] can be considered the last one, fulfilling three items (100% methodological). If a weight is assigned to the methodological items (the last 7: causal modelling ones), for values greater than 0.51, the new ranking is: [61], [57,60], [22,58], [59], and finally [62].

### 3.5. Implications for Policy-Making in Mental Health Care

All studies included in the present systematic review developed a causal model that can be used, directly or indirectly, for guiding MHSS management and planning, but only two were designed to be integrated in a DSS [22,61].

Regarding studies that assessed a single MHSS, recommendations for shelter home services focused on the finding that the interaction between the organizational structure of the services (shelter homes) and the community (real environment) should be improved because it has a relevant impact on the service delivered to homeless people [59]. In addition, integrated care should be provided within the community to avoid the segregation of homeless people, and it is crucial to carry out specialized training in this population’s needs for social workers. In the case of nursing home services, it may be possible to decrease the wrong placement capacity by reorganizing the process of treatment in general hospitals and providing training for families who would like to take care of patients at home, being the admission policy determinant, especially for elderly people to mental hospitals [57].

Following with studies that assessed a group of MHSSs, the results suggested that a reduction of beds, based on the deinstitutionalization policy, had a variable impact, depending on the mental disorders, on hospital admissions (e.g., decreasing depression admissions and increasing eating disorders admissions), and on length of stay (e.g. significant decreasing for specific diagnoses such as abuse of alcohol) [62]. Moreover, evidence shows that the changes in admissions are not associated with the activity of community mental health teams after deinstitutionalization. Additionally, the relationship between patient needs and outcomes was partially mediated by service performance, which means that improving mental health service performance is important for improving recovery outcomes [58]. The effectiveness of MHSS for people with the highest needs is lower, and the main implications showed the importance of developing recovery-oriented services (e.g., assertive community treatment, intensive case management, and supported employment) for helping this kind of user; consequently, increased investment in specialist services is needed.

On the other hand, only one study include service-user information that can be considered an approximation to the analysis of service user perspectives: quality of life and personal recovery [58]. Service user experiences and family opinions are relevant clinical outcomes for assessing service performance and quality, as well as in designing mental health interventions and policies.

## 4. Discussion

To the best of our knowledge, this is the first study to collect empirical evidence of causal modelling applications (BNs and SEM) for MHSS planning and management. It followed the PRISMA guidelines [48] by designing an integrative and extensive search strategy without restrictive inclusion criteria. This article provides an updated state of the art and highlights some strategies for policy-making.

Although causal modelling has been widely applied in general health and mental health care for supporting decision-making (patient level, e.g., for supporting the diagnosis) [35,37,38], it is not the case for the other levels of analysis (micro, meso, and macro). Despite its utility and increased interest in the last years [22,58,61,62], the development of causal modelling is still scarce due to the complexity (the number of variables –sometimes grouped in imprecise domains or constructs- and their causal relationships –sometimes difficult to explain- are very high) and the uncertainty (the statistical nature of the variables are unknown –unreliable or imprecise- and there are missing variables) of real environments. As it was stated before, it is very difficult to identify the critical variables/domains and their causal relationships without formal expert-based models that can explain the behaviour of mental health systems.

The results show that causal models are accurate for supporting the management of not only specialized MHSS, but also nursing homes, secure medium services, and shelter homes that provide care for mentally ill people. In conclusion, advanced and hybrid methodologies are appropriate tools for supporting decision-making, planning, and management of mental health services [22,61]. Regarding the services and organization, the performance of mental health services plays a role in recovery outcomes and care provision [58], as well as the interaction between the environment and shelter homes impact on care provision to homeless users [59]. In this line, organizational interventions, such as reductions in bed availability, impact on service utilization [62]. In addition, socioeconomic and demographic factors explained the utilization of long-term care services [57]. In addition, it was shown how process management impacted hospital performance, and this information can be used by planners and managers of mental hospitals for decision-making [60]. Finally, although three studies [22,58,62] provided MHSS information from a holistic perspective, integrating hospital and community-based care, there was a lack of systematization of mental health care provision.

The new extensive checklist for quality assessment of the selected studies was sufficiently discriminative, and only one criterion should be considered arguable (all the studies fulfilled it). Taking into account that the checklist included both structural (MHSS) and methodological (causality) questions, it can be considered as a basis for new proposals in this research field. By using the checklist, it is possible to rank the selected studies according to their quality.

This paper also notes the extreme variability in the terminology used for MHSSs (e.g., medium secure service, shelter organizations, mental hospitals, community-based mental health agencies, community mental health housing resources, or nursing homes). Just one out of seven studies [22] used an international standard codification tool called DESDE-LTC [51],for classifying the mental health systems according to the main type of care provided (residential, day, or outpatient care). In addition, this book chapter included a large sample of MHSSs, grouped in small health areas, providing a meso-level analysis. The lack of using standard classification systems, such as DESDE-LTC or ESMS [53], is a handicap to making international comparisons or conducting meta-analysis among studies, catchment areas, and services [63,64,65].

### Limitations

The high variability of the included studies did not allow us to carry out a meta-analysis. In addition, the lack of mental health services standardization did not make it possible to compare MHSS internationally. There is no standardized quality assessment tool for this area of study, which can be considered another limitation. This limitation was overcome by developing an ad hoc checklist.

## 5. Conclusions

In the present study, it is stated that, in spite of the potential utility, there are few studies that have applied causal modelling for supporting MHSS planning and management. Causal modelling utility is demonstrated by checking the variability of the systems under study. By applying causal modelling, it is possible to identify relevant strategies in policy-making. Finally, it is feasible to assess the quality of the studies by using the checklist developed in this paper. 

Therefore, keeping in mind the current context characterized by economic constrains and gaps of unmet population needs [11,12,13,14], MHSS planning and management should be dramatically improved because they have a key role in decreasing the gaps and increasing the MHSS efficiency and effectiveness. MHSS research is crucial for designing evidenced-informed decisions, which will improve care delivery for people suffering from a mental disease. In this sense, it is highly recommended that new studies are developed to identify causal relationships among the different elements of the mental health care system for guiding decision-making. Following with this idea, the inclusion of service user perspectives as clinical outcomes is essential to designing new BNs (they can be causes or effects, depending on the causal model orientation) for mental health management and planning.

## Figures and Tables

**Table 1:** Acronyms used in the text.

Acronym	Description
MHSS	Mental health services and systems
DSS	Decision support system
BN	Bayesian network
SEM	Structural Equation Modelling
PRISMA	Preferred Reporting Items for Systematic Reviews and Meta-Analyses
PROSPERO	International Prospective Register of Systematic Reviews
ICC	Intra-Class Correlation
B-MHCC	Basic Mental Health Community Care
AUC	Area Under the Curve
MBNQA	Malcolm Baldrige National Quality Award
DEA	Data Envelopment Analysis
BCC	Returns variables to scale

**Table 2:** Search strategy piloted in MEDLINE–PubMed version (First version).

Identifies All Types of Mental Health Services [Title/Abstract]
1. "Mental health"
2. "Mental disorder*"
3. "Mental illness*"
4. "Psychiatric disorder*"
5. "Psychopathology"
6. 1 OR 2 OR 3 OR 4 OR 5
Identifies methods for causality assessment [Title/Abstract]
7. "Bayesian network*"
8. "Causal model"
9. "Causal reasoning"
10. 6 OR 7 OR 8 OR 9
11. 6 AND 10

**Table 3:** Search strategy piloted in MEDLINE–PubMed version.

Identifies All Types of Mental Health Services [Title/Abstract]
1. "Mental health"
2. "Mental health care"
3. "Mental health service*"
4. "Mental health system*"
5. "Psychiatric care"
6. "Psychiatric hospital*"
7. "Inpatient care"
8. "Residential Care"
9. "Outpatient care"
10. "Day care"
11. "Community mental health cent*"
12. "Residential facilit*"
13. "Residential service*"
14. "Assisted living facilit*"
15. "Halfway house*"
16. "Nursing home*"
17. "Support* accom*"
18. "Support* tenanc*"
19. "Floating support"
20. "Floating outreach"
21. 1 OR 2 OR 3 OR 4 OR 5 OR 6 OR 7 OR 8 OR 9 OR 10 OR 11 OR 12 OR 13 OR 14 OR 15 OR 16 OR 17 OR 18 OR 19 OR 20
Identifies methods for causality assessment [Title/Abstract]
22. "Bayesian network*"
23. "Structural equation*"
24. "Causal model*"
25. "Causal reasoning"
26. 22 OR 23 OR 24 OR 25
Identifies terms of management and planning [All fields]
27. "Manag*"
28. "Decision Support"
29. "Decision making"
30. "Expert knowledge"
31. "Planning"
32. 27 OR 28 OR 29 OR 30 OR 31
33. 21 AND 26 AND 32

**Table 4:** Results: Study characteristics.

Authors, Year and Country	Objectives	Type of MHSS	Target Population	Data	Variables (Scale)	Methods
Constantinou et al., 2015, United Kingdom	To develop a decision support system for violence risk assessment and risk management in patients discharged from medium secure services (DSVM-MSS).	Medium secure services that provide accommodation, support, and treatment.	Patients with mental health problems. Total of 386 patients discharged from medium secure services. Total of 953 prisoners, of whom 594 are mentally ill (anger management, drug misuse treatment, alcohol misuse treatment, cocaine dependence, cannabis dependence, stimulants dependence, and alcohol dependence). All of them are 18 years old or older.	Datasets were collected from The Validation of New Risk Assessment Instrument for Use with patients Discharged from Medium Secure Service, Prisoner Cohort Study, and criminal records retrieved by the Police National Computer.	IQ, Structured leisure activities, Stable and suitable work, Effective coping skills, Steady income, Positive life goals, Pro-social and supportive network, Professionally supervised living, Problems with intimate relationships, Problems with other relationships, Problems with employment, Social avoidance, Self-control, Inadequate planning, Personal resources, Expert, Delusions, Hallucinations, Anxiety, Depression, Grandiosity, Psychotic illness, Cannabis use, Cannabis use post treatment, Cocaine use, Cocaine use posttreatment, Heroin use, Stimulants use, Stimulants use posttreatment, Opiates use, Hazardous drinking, Alcohol treatment, Hazardous drinking posttreatment, Drug treatment, Cannabis dependence, Cocaine dependence, Heroin dependence, Stimulants dependence, Opiates dependence, Alcohol dependence, Substance dependence, Disinhibition, Excessive substance use, Personality disorder, PCLSV factor 1, PCLSV factor 2, PCLSV facet 3, Poor parenting, Secure attachment in childhood, Instability, Problems with ASB as adult, Motivation for treatment, Motivated to use medication, Uncooperativeness, Negative attitude, Problems with responsiveness, Lack of insight, Medication at discharge, Tension, Guilt feelings, Affective lability, Anger, Anger management, Anger posttreatment, Excitement, Suspiciousness, Hostility, Difficulty delaying gratification, Emotional withdrawal, Aggression, Uncontrolled aggression, Gender, Age, Length of stay as inpatient, Pro-criminal attitude, Victimization, Violent ideation or intend, Serious problems with violence, Prior serious offences, General violence and Violent convictions. All group 2: Service user´s characteristics.	Bayesian network (BN): expert knowledge for constructing the causal structure of the (BN), binary factors and combinatorial rules, conditional probability tables, expectation maximization algorithm, graph surgery, area under the curve (AUC) of a receiver operating characteristic measure, leave-one-out cross-validation, causal-related inference, T-test, and sensitivity analysis (tornado graphs).
Delany et al., 1994, United States of America	To develop a model to test the effect of organizational structure on organizational relations and on services and amenities, both of them being mediated by organizational relations.	A total of 192 shelter organizations that provided overnight accommodation; health, substance abuse, and mental health services in 29 cities in the continental United States.	Service users without access to adequate and usual accommodations.	Data were collected using a survey questionnaire sent to shelter directors or managers. The questionnaire included information about organizational funding, affiliation, mission, and target population; relationships with groups in the community; perceptions of the stability of the environment; obstacles to operation (zoning, health code issues, lack of transportation); and operational policies, including level of formalization and centralization, staffing patterns, problems with staff, staff autonomy and routine, and services and amenities.	Organizational structure: formalization, autonomy, specialization, routinization, knowledge complexity, and centralization. All group 1: Resources. Organizational relations: diversity of funding, relationships, constraints, and independence. All group 1: Resources. Services and amenities: Personal maintenance needs, case management services, and health substance abuse and mental health services. All group 1: Resources.	Structural Equation Modelling (SEM), covariance analysis, Bentler-Bonnet Index, Z-score test, and confirmatory factor analysis.
Green and Griffiths, 2014, United Kingdom	To analyze trends in hospital and community treatment in England.	Mental health services of NHS England: NHS hospitals, NHS funded beds in independent hospitals, NHS mental health teams, community crisis teams, and community psychiatric services.	Adults diagnosed with eight severe diagnoses: schizophrenia (F20), bipolar affective disorder (31), depressive disorder (F32), recurrent depressive disorder (F33), eating disorder (F50), mental and behavioural disorder due to use of alcohol (F10), unspecified dementia (F03), and reaction to stress and adjustment disorders (F43), according to ICD-10 diagnostic categories.	Data were collected from 1998 to 2012 across NHS England from the UK Government Health and Social Care Information Centre, the published Health Episode Statistics spreadsheets on primary diagnosis of admissions, the annual open records of community crisis team in England from 2003 to 2010, and the UK Department of Health.	Annual numbers of available hospital beds Group 1: Resources. Eight ICD-10 adult mental disorder Group 2: Service user’s characteristics. Hospital admissions, median length of stay, annual numbers of Mental Health Act detentions, and community team activity. Group 3: Service performance and outcomes.	Linear regression, Pearson’s r-statistic, SEM, parametric bootstrap, and two-tailed t test.
Kim and Oh, 2012, Korea	To develop health care evaluation criteria for mental health care according to the Malcolm Baldrige National Quality Award model (MBNQA).	Five state-operated mental hospitals in Korea.	Service users of the hospitals under analysis.	Authors developed a survey based on the MBNQA and previous findings. The survey was directed to physician, nurses, medical technicians, pharmacists, and administrative staff at the five state-operated hospitals across Korea.	Driver: Leadership. Direction: Strategic planning. System: Human Resources Orientation; Process Management; and Patient, customer, & Market Orientation. Foundation: Measurement, Analysis, & Knowledge management. Group 1: Resources. Results: Hospital Performance. Group 3: Service performance and outcomes.	Confirmatory factor analysis and SEM analysis.
Roux et al., 2016, Canada	To analyze the role of service performance as a mediating factor between severity of patient’s needs and outcomes.	Mental health service networks from Quebec, including the hospital department of psychiatry, multidisciplinary mental health primary care team, community-based mental health agencies, general practitioners and psychologists practicing in private clinics, and community mental health housing resources.	Adults from 18 to 70 years old, diagnosed with schizophrenia spectrum disorders, mood, anxiety, obsessive-compulsive, personality, attention-deficit hyperactivity, or stressor-related disorders, according to DSM-5 diagnostic categories.	Datasets were collected from five questionnaires: Montreal Assessment of Needs Questionnaire (MANQ), Alberta Continuity of Services Scale for Mental Health, Recovery Self-Assessment Scale, Satisfaction with Life Domains Scale, and Recovery Assessment Scale.	Needs: Intensity of needs (Montreal Assessment of Needs Questionnaire). Group 2: Service user’s characteristics. Service performance: Adjusted adequacy of help (Montreal Assessment of Needs Questionnaire), Continuity of care (Alberta Continuity of Services Scale for Mental Health), and Recovery service orientation (Recovery Self-Assessment Scale, revised person-in-recovery version). Outcomes: Quality of life (Satisfaction with Life Domains Scale) and Personal recovery (Recovery Assessment Scale). Group 3: Service performance and outcomes.	Zero order correlations, Pearson’s correlations, bootstrap method with 2000 iterations; SEM and mediation analysis. Factor loadings, regression analyses, non-parametric model-based bootstrapping with 2000 iterations, chi-squared goodness-of-fit statistic, Bollen-Stine bootstrap method, Tucker-Lewis Index, and root-mean-square error of approximation.
Salvador-Carulla et al., 2013, Spain	To improve the relative technical efficiency assessment by establishing causal relationships among variables.	Seventy-one small mental health areas in Andalucía (Spain). The main type of care provided, according to the ESMS/DESDE-LTC coding, is acute and non-acute care (hospital), residential nonhospital care, day acute and non-acute care, and other structured activities.	Adults who had experienced mental disorders.	Datasets were retrieved by The Public Mental Health System of Andalusia (Spain).	Public health budget, professional workers, and accessibility. Group 1: Resources. Risks factors for mental health and psychiatric morbidity. Group 2: Service user’s characteristics. Treated prevalence in a small health area in a specific year t (patients_t), patients already in contact with mental health community service in the year t-1 (patients_t-1), new patients who contact the specialized community services in this year (new patients_t), activities with patients, and relative technical efficiency. Group 3: Service performance and outcomes.	“Bayesian network Data Envelopment Analysis model”: Data Envelopment Analysis (DEA) with returns variables to scale (BCC), BN integrating fuzzy rules base to interpret causal relationships, interpretation of efficiency variables according to rule-base “if…then” (Model of Basic Mental Health Community Care). Services are standardized using ESMS/DESDE-LTC classification system.
Wolf, 1978, United States of America	To analyze the effect of sociocultural and health-resource variables on long-term-care utilization.	Thirty-nine mental health catchment areas of Massachusetts, including 901 nursing homes. The 901 nursing homes housed 49,471 residents. The 901 nursing homes included 38 chronic disease and rehabilitation hospitals, which provided accommodation for 5803 service users. Hospital care, care in general hospitals, and nursing home care.	Patients who lived in the catchment area where the facilities were located. Patients 65 and older who were admitted to Massachusetts Department Mental Health, discharged from general hospitals, of home health care programs, and patients 60 and older in nursing homes and chronic disease and rehabilitation hospitals.	Datasets were collected from the Commonwealth of Massachusetts, the state-wide survey for assessing community programs sponsored by the Massachusetts Department of Mental Health, and the survey conducted by the Massachusetts Department of Public Health.	Community Care Resources and Primary Care Resources. Group 1: Resources. Socioeconomic Status, Marital Status/Living Arrangement, Age, Ethnicity, Race, and Urbanization. Group 2: Service user’s characteristics. Mental Hospital Utilization, General Hospital Utilization, and Long-Term Care Utilization. Group 3: Service performance and outcomes.	Path analysis, path coefficients, test of variable distributions for normality, regression analysis, factor analysis, covariance analysis, path diagram, zero-order correlations, and multiple regression equations.

**Table 5:** Results: Main Findings.

Study	Complexity	Main Findings
Constantinou et al., 2015	Single MHSS Micro level	1. The decision support system “DSVM-MSS” predicted general violence (area under the curve scores = 0.691 (pre-discharge) and 0.730 (post-discharge); this difference is not statistically significant (*p* = 0.472)) and violent convictions (area under the curve scores = 0.845 (pre-discharge) and 0.774 (post-discharge); this difference was not statistically significant (*p* = 0.469)) in people with mental health problems living in medium secure services.
Delany et al., 1994	Single MHSS Micro level	1. The direct relationship between organizational structure (*formalization, autonomy, specialization, routinization, knowledge complexity*, and *centralization*) and service amenities *(personal maintenance needs, case management services*, and *health substance abuse and mental health services*) was not statistically significant (*z* = 0.363). 2. The direct relationship between organizational structure and organizational relations (*diversity of funding, relationships, constraints*, and *independence*) was statistically significant beyond the 0.01 level (*z* = 3.152). 3. The direct relationship between organizational relations and services amenities was not statistically significant (*z* = 1.482). 4. The organizational structure affected services and amenities (personal maintenance needs, case management services, and health-substance abused and MHS) through the organizational relations dimension, including funding, relationships, constrains, and independence. 5. The model showed a good reproduction of the observed covariance matrix for the following variables: *specialization*; *diversity of funding; relationships*; *constrains*; *personal maintenance needs*; *case management services*; and *health, substance abuse*, *and mental health services*: ξ^2^ (11) = 18.908, *p* = 0.06275; Bentler-Bonnet Fit Index = 0.84.
Kim and Oh, 2012	Single MHSS Micro level	1. *Leadership* positively and significantly (*p* = 0.000) impacted *Measurement, Analysis*, and *Knowledge Management*; *Strategic Planning*; *Patient, Customer*, and *Market Orientation; and Human Resources Orientation*. *Leadership* did not significantly impact *Process Management* (*p* = 0.574) or *Hospital Performance* (*p* = 0.190). 2. *Strategic Planning* positively and significantly (*p* = 0.000) affected *Patient*, *Customer*, and *Market Orientation* and *Process Management*(*p* = 0.004), and it did not impact significantly on *Human Resources Orientation* (*p* = 0.492). 3. *Patient*, *Customer*, and *Market Orientation* positively and significantly impacted *Hospital Performance* (*p* = 0.000) and *Process Management* (*p* = 0.017). 4. *Human Resources Orientation* impacted *Process Management* (*p* = 0.000) and *Hospital Performance* (*p* = 0.000). 5. *Process Management* positively influenced *Hospital Performance* (*p* = 0.000). 6. *Measurement*, *Analysis*, and *Knowledge Management* positively impacted *Strategic Planning*; *Patient*, *Customer*, and *Market Orientation*; *Human Resources Orientation*; and *Process Management* (*p* = 0.000). 7. The structural model showed the following results: *χ*^2^ = 14.034 (*df* = 3), *p* = 0.012, *χ*^2^/*df* = 4.678, Goodness-of-fit Index = 0.994, Root Mean Residual = 0.009, Normed Fit Index = 0.997, and Confirmatory Fit Index = 0.998.
Wolf, 1978	Single MHSS Micro and Meso levels	1. Mental hospital utilization had a weak and negative impact on long-term utilization of nursing homes (*r* = −0.071). 2. Catchment areas where there are more admissions of elderly people had a higher percentage of urban (*β* = −0.089), non-white (*β* = 0.074), aged persons (*β* = 0.105) and more persons unmarried and living alone (*β* = 0.160). The proportion of foreign-born people did not influence the model (*β* = 0.001). This model explained 9% of the variance in mental hospital utilization.
Green and Griffiths, 2014	Group of MHSSs Micro level	1. The reduction of beds availability entailed an annual inpatient admissions decrease in: depression (*β* = −1085; *p* < 0.01), dementia (*β* = −764; *p* < 0.01), schizophrenia (*β* = −468; *p* < 0.01), bipolar disorder (*β* = −159; *p* < 0.01), and OCD (*β* = −21; *p* < 0.01); and increase in use of alcohol (*β* = 1764; *p* < 0.01), eating disorders (*β* = 55; *p* < 0.01), and posttraumatic stress disorder (*β* = 17; *p* < 0.01). 2. The reduction of beds availability significantly decreased length of hospital stay in: use of alcohol (*β* = −0.29, *p* < 0.001), eating disorders (*β* = −0.52, *p* < 0.001), dementia (*β* = −0.55, *p* < 0.001), and depression (*β* = −0.96, *p* < 0.001). 3. The reduction of beds availability increased the number of detentions under Mental Health Act (*β* = 298, *p* < 0.01). 4. The number of mental health beds was negatively associated with the number of psychiatric severe admissions (coefficient = −0.683; *p* < 0.001, bootstrapped 95% CI: 0.37 to 1.06). 5. The number of beds was negatively associated with community team activity (coefficient = −0.521; *p* < 0.001, bootstrapped 95% CI: −0.71 to 0.25). 6. The community team activity was not associated with inpatient admissions (coefficient = −0.121, *p* < 0.001, bootstrapped 95% CI: −0.35 to 0.42). 7. The model (a path from community team activity to hospital beds and from hospital beds to hospital admissions) showed good fit: *χ*^2^ = 0.57; *df* = 1; *p* = 0.45; Tucker–Lewis Index = 1.07, root mean square error of approximation = 0.00.
Roux et al., 2016	Group of MHSSs Micro level	1. Patient needs (*adaptation to stress*, *social exclusion*, *involvement in treatment decisions*, and *job integration*) and outcomes (*quality of life* and *personal recovery*) were negatively associated (*β* = −0.60; *p* < 0.001). 2. Service performance (*type* and *amount of support* provided) and outcomes were positively associated (*β* = 0.40; *p* < 0.001). 3. Patient needs and service performance were negatively associated (*β* = −0.30; *p* < 0.001). 4. The model provided a good fit for the data, as suggested by the following statistics: non-significant goodness-of-fit based on the Bollen–Stine bootstrap distribution ((7) = 14.3, *p* = 0.107), TLI above 0.95 (TLI = 0.967) and RMSEA not statistically greater than 0.05 (RMSEA = 0.056, one-sided P = 0.358). The model explained 67% of the variance in outcomes. 5. Service performance had a partial mediation role between needs and outcome. A total of 16.4% of the impact of needs on outcomes was mediated by service performance (standard error: 0.05, *z* = 3.6, p < 0.001 with the Bollen–Stine bootstrap method after 2000 iterations).
Salvador-Carulla et al., 2013	Group of MHSSs Meso level	1. The treated prevalence of a small health area during a specific year was the result of combining service users that were in contact with the mental health service (during the year t-1) and the new services users who contacted the specialized mental health services within this year. Psychiatric morbidity was the root variable, which caused the treated incidence of new patients and the treated prevalence of patients who were in contact with mental health community services. Treated prevalence directly influenced workforce capacity, relative technical efficiency, and activities with patients. Another root variable is public health budget, directly related to workforce capacity. Accessibility was the third root variable that influenced the treated incidence of new patients.

**Table 6:** Checklist for quality assessment

Quality Assessment Statements	Constantinou et al., 2015	Delany et al., 1994	Kim and Oh, 2012	Green and Griffiths, 2014	Roux et al., 2016	Salvador-Carulla et al., 2013	Wolf, 1978
1. Includes more than one type of mental health service or system				X	X	X	
2. Specifies more than one type of target population for care delivery				X	X	X	
3. Variables include resources and outcomes of the mental health care	X	X	X	X	X	X	X
4. Includes a causal graph	X	X	X		X	X	X
5. Takes into account external expert knowledge for identifying the nodes and the causal relationships of the causal graph	X		X			X	X
6. Combines data and external expert-based knowledge	X		X		X		X
7. Include sensitivity or parametric analysis	X	X		X	X		
8. Carries out factorial confirmatory/exploratory analysis		X	X				X
9. Develops causal-related inference	X			X			
10. The causal model is integrated in a decision support system	X					X	

## References

[1] Thornicroft G., Tansella M. (2009). Better Mental Health Care.

[2] Thornicroft G., Tansella M. (1998). A conceptual framework for mental health services: The matrix model. Psychol. Med..

[3] Thornicroft G., Szmukler G., Mueser K.T., Drake R.E. (2011). Oxford Textbook of Community Mental Health.

[4] Bouras N., Ikkos G., Craig T. (2018). From Community to Meta-Community Mental Health Care. Int. J. Environ. Res. Public Health.

[5] Gibert K., García-Alonso C., Salvador-Carulla L. (2010). Integrating clinicians, knowledge and data: Expert-based cooperative analysis in healthcare decision support. Health Res. Policy Syst..

[6] Salvador-Carulla L., Haro J.M., Ayuso-Mateos J.L. (2006). A framework for evidence-based mental health care and policy. Acta Psychiatr. Scand..

[7] Vigo D., Thornicroft G., Atun R. (2016). Estimating the true global burden of mental illness. Lancet Psychiatry.

[8] Bloom D.E., Cafiero E., Jané-Llopis E., Abrahams-Gessel S., Reddy Bloom L., Fathima S.B., Feigl A., Gaziano T., Hamandi A., Mowafi M. (2011). The Global Economic Burden of Noncommunicable Diseases. World Econ. Forum.

[9] Trautmann S., Rehm J., Wittchen H.-U. (2016). The economic costs of mental disorders: Do our societies react appropriately to the burden of mental disorders?. EMBO Rep..

[10] Collins P., Patel V., Joestl S., March D., Insel T., Daar A., Anderson W., A Dhansay M., Phillips A., Shurin S. (2011). Grand Challenges in Global Mental Health. Nature.

[11] World Health Organization (2013). Mental Health Action Plan 2013-2020.

[12] Pathare S., Brazinova A., Levav I. (2018). Care gap: A comprehensive measure to quantify unmet needs in mental health. Epidemiol. Psychiatr. Sci..

[13] Thornicroft G. (2007). Most people with mental illness are not treated. Lancet.

[14] Alonso J., Codony M., Kovess V., Angermeyer M.C., Steven J., Haro J.M., Girolamo G.D.E., Graaf R.O.N.D.E., Demyttenaere K., Vilagut G. (2007). Population level of unmet need for mental healthcare in Europe service AUTHOR ’ S PROOF Population level of unmet need for mental healthcare in Europe *. Br. J. Psychiatry.

[15] Andrews G., Cuijpers P., Craske M.G., McEvoy P., Titov N. (2010). Computer Therapy for the Anxiety and Depressive Disorders Is Effective, Acceptable and Practical Health Care: A Meta-Analysis. PLoS ONE.

[16] Christensen H., Pallister E., Smale S., Hickie I.B., Calear A.L. (2010). Community-Based Prevention Programs for Anxiety and Depression in Youth: A Systematic Review. J. Prim. Prev..

[17] Sin J., Gillard S., Spain D., Cornelius V., Chen T., Henderson C. (2017). Effectiveness of psychoeducational interventions for family carers of people with psychosis: A systematic review and meta-analysis. Clin. Psychol. Rev..

[18] Correl C.U., Galling B., Pawar A., Krivko A., Boneto C., Ruggeri M., Craig T., Nordentoft M., Srihari V., Guloksuz S. (2018). Comparison of early intervention services vs treatment as usual for early-phase psychosis: A systematic review, meta-analysis, and meta-regression. JAMA Psychiatry.

[19] Silvestri F., Peters J. (2007). An Introduction to the International Initiative for Mental Health Leadership (IIMHL). Int. J. Leadersh. Public Serv..

[20] Beinecke R.H., Daniels A., Peters J., Silvestri F. (2009). Guest Editors’ Introduction: The International Initiative for Mental Health Leadership (IIMHL): A Model for Global Knowledge Exchange. Int. J. Ment. Health.

[21] Salvador-Carulla L., Amaddeo F., Gutiérrez-Colosía M.R., Salazzari D., Gonzalez-Caballero J.L., Montagni I., Tedeschi F., Cetrano G., Chevreul K., Kalseth J. (2015). Developing a tool for mapping adult mental health care provision in Europe: The REMAST research protocol and its contribution to better integrated care. Int. J. Integr. Care.

[22] Salvador-Carulla L., Garcia-Alonso C., Gibert K., Vázquez-Bourgon J. (2013). Incorporating local information and prior expert knowledge to evidence-informed mental health system research. Improving Mental Health Care.

[23] Constantinou A.C., Fenton N., Marsh W., Radlinski L. (2016). From complex questionnaire and interviewing data to intelligent Bayesian network models for medical decision support. Artif. Intell. Med..

[24] Torres-Jiménez M., García-Alonso C.R., Salvador-Carulla L., Fernández-Rodríguez V. (2015). Evaluation of system efficiency using the Monte Carlo DEA: The case of small health areas. Eur. J. Oper. Res..

[25] Pearl J. (2009). Causality: Models, Reasoning and Inference.

[26] Constantinou A.C., Fenton N., Neil M. (2016). Integrating expert knowledge with data in Bayesian networks: Preserving data-driven expectations when the expert variables remain unobserved. Expert Syst. Appl..

[27] Greenland S., Pearl J., Robins J.M. (1999). Causal diagrams for epidemiologic research. Epidemiology.

[28] Yet B., Bastani K., Raharjo H., Lifvergren S., Marsh W., Bergman B. (2013). Decision support system for Warfarin therapy management using Bayesian networks. Decis. Support Syst..

[29] Topuz K., Zengul F.D., Dag A., Almehmi A., Bayram M. (2018). Predicting graft survival among kidney transplant recipients: A Bayesian decision support model. Decis. Support Syst..

[30] Lin J., Haug P.J. (2008). Exploiting missing clinical data in Bayesian network modeling for predicting medical problems. J. Biomed. Inform..

[31] Cashion A.K., Hathaway D.K., Stanfill A., Thomas F., Ziebarth J.D., Cui Y., Cowan P.A., Eason J. (2014). Pre-transplant predictors of one yr weight gain after kidney transplantation. Clin. Transplant..

[32] Curiac D., Vasile G., Banias O., Volosencu C., Albu A. Bayesian Network Model for Diagnosis of Psychiatric Diseases. Proceedings of the ITI 2009 31st International Conference on Information.

[33] McNally R.J. (2016). Can network analysis transform psychopathology?. Behav. Res. Ther..

[34] Sorias S. (2015). Overcoming the limitations of the descriptive and categorical approaches in psychiatric diagnosis: A proposal based on bayesian networks. Turk Psikiyatr. Derg..

[35] Estabragh Z.S., Mansour M., Kashani R., Moghaddam F.J., Sari S. Bayesian Network Model for Diagnosis of Social Anxiety Disorder. Proceedings of the 2011 IEEE International Conference on Bioinformatics and Biomedicine Workshops (BIBMW).

[36] Chang Y., Hung W., Juang T. Depression Diagnosis based on Ontologies and Bayesian Networks. Proceedings of the 2013 IEEE International Conference on Systems, Man, and Cybernetics.

[37] Ojeme B., Mbogho A. (2016). Selecting Learning Algorithms for Simultaneous Identification of Depression and Comorbid Disorders. Procedia Comput. Sci..

[38] Seixas F.L., Zadrozny B., Laks J., Conci A., Muchaluat Saade D.C. (2014). A Bayesian network decision model for supporting the diagnosis of dementia, Alzheimer’s disease and mild cognitive impairment. Comput. Biol. Med..

[39] Pinheiro P.R., De Castro A.K.A., Pinheiro M.C.D. A Multicriteria Model Applied in the Diagnosis of Alzheimer’s Disease: A Bayesian Network. Proceedings of the 2008 11th IEEE International Conference on Computational Science and Engineering.

[40] Goodson J., Jang W. (2008). Assessing nursing home care quality through Bayesian networks. Health Care Manag. Sci..

[41] World Health Organization (2016). mhGAP Intervention Guide for Mental, Neurological, and Substance Use Disorders in Non-Specialized Health Settings. Version 2.0.

[42] Joint Commissioning Panel for Mental Health (2012). Rehabilitation Services for People with Complex Mental Health Needs.

[43] Dieterich M., Irving C.B., Bergman H., Khokhar M.A., Park B., Marshall M. (2017). Intensive case management for severe mental illness (review). Cochrane Database Syst. Rev..

[44] Tansella M., Thornicroft G., Lempp H. (2014). Lessons from community mental health to drive implementation in health care systems for people with long-term conditions. Int. J. Environ. Res. Public Health.

[45] Marshall M., Lockwood A. (2000). Assertive community treatment for people with severe mental disorders (Review). Cochrane Database Syst. Rev..

[46] McKay C., Nugent K.L., Johnsen M., Eaton W.W., Lidz C.W. (2016). A Systematic Review of Evidence for the Clubhouse Model of Psychosocial Rehabilitation. Adm. Policy Ment. Heal. Ment. Heal. Serv. Res..

[47] Salvador-Carulla L., García-Alonso C.R., González-Caballero J.L., Garrido-Cumbrera M. (2007). Use of an operational model of community care to assess technical efficiency and benchmarking of small mental health areas in Spain. J. Ment. Health Policy Econ..

[48] Moher D., Liberati A., Tetzlaff J., Altman D.G., Altman D., Antes G., Atkins D., Barbour V., Barrowman N., Berlin J.A. (2009). Preferred reporting items for systematic reviews and meta-analyses: The PRISMA statement (Chinese edition). J. Chin. Integr. Med..

[49] Priebe S., Saidi M., Want A., Mangalore R., Knapp M. (2009). Housing services for people with mental disorders in England: Patient characteristics, care provision and costs. Soc. Psychiatry Psychiatr. Epidemiol..

[50] Killaspy H., Priebe S., Bremner S., McCrone P., Dowling S., Harrison I., Krotofil J., McPherson P., Sandhu S., Arbuthnott M. (2016). Quality of life, autonomy, satisfaction, and costs associated with mental health supported accommodation services in England: A national survey. Lancet Psychiatry.

[51] Salvador-Carulla L., Alvarez-Galvez J., Romero C., Gutiérrez-Colosía M.R., Weber G., McDaid D., Dimitrov H., Sprah L., Kalseth B., Tibaldi G. (2013). Evaluation of an integrated system for classification, assessment and comparison of services for long-term care in Europe: The eDESDE-LTC study. BMC Health Serv. Res..

[52] Montagni I., Salvador-Carulla L., Mcdaid D., Straßmayr C., Endel F., Näätänen P., Kalseth J., Kalseth B., Matosevic T., Donisi V. (2017). The REFINEMENT Glossary of Terms: An International Terminology for Mental Health Systems Assessment. Adm. Policy Ment. Heal. Ment. Heal. Serv. Res..

[53] Johnson S., Kuhlmann R. (2000). The European Service Mapping Schedule (ESMS): Development of an instrumentfor the description and classificationof mental health services. Acta Psychiatr. Scand..

[54] Pearl J. (2010). An Introduction to Causal Inference. Int. J. Biostat..

[55] Killaspy H., White S., Dowling S., Krotofil J., McPherson P., Sandhu S., Arbuthnott M., Curtis S., Leavey G., Priebe S. (2016). Adaptation of the Quality Indicator for Rehabilitative Care (QuIRC) for use in mental health supported accommodation services (QuIRC-SA). BMC Psychiatry.

[56] Thomas B.H., Ciliska D., Dobbins M., Micucci S. (2004). A process for systematically reviewing the literature: Providing the research evidence for public health nursing interventions. Worldviews Evid.-Based Nurs..

[57] Wolf R.S. (1978). A social systems model of nursing home use. Health Serv. Res..

[58] Roux P., Passerieux C., Fleury M.-J. (2016). Mediation analysis of severity of needs, service performance and outcomes for patients with mental disorders. Br. J. Psychiatry.

[59] Delany P.J., Fletcher B.W., Lennox R.D. (1994). Analyzing shelter organizations and the services they offer: Testing a structural model using a sample of shelter programs. Eval. Progr. Plann..

[60] Kim Y.K., Oh H.J. (2012). Causality analysis on health care evaluation criteria for state-operated mental hospitals in Korea using Malcolm Baldrige National Quality Award Model. Community Ment. Health J..

[61] Constantinou A.C., Freestone M., Marsh W., Coid J. (2015). Causal inference for violence risk management and decision support in forensic psychiatry. Decis. Support Syst..

[62] Green B.H., Griffiths E.C. (2014). Hospital admission and community treatment of mental disorders in England from 1998 to 2012. Gen. Hosp. Psychiatry.

[63] Sadeniemi M., Almeda N., Salinas-Pérez J.A., Gutiérrez-Colosía M.R., García-Alonso C., Ala-Nikkola T., Joffe G., Pirkola S., Wahlbeck K., Cid J. (2018). A Comparison of Mental Health Care Systems in Northern and Southern Europe: A Service Mapping Study. Int. J. Environ. Res. Public Heal..

[64] Salvador-Carulla L., Tibaldi G., Johnson S., Scala E., Romero C., Munizza C. (2005). Patterns of mental health service utilisation in Italy and Spain. An investigation using the European Service Mapping Schedule. Soc. Psychiatry Psychiatr. Epidemiol..

[65] Gutierrez-Colosia M.R., Salvador-Carulla L., Salinas-Perez J.A., Garcia-Alonso C.R., Cid J., Salazzari D., Montagni I., Tedeschi F., Cetrano G., Chevreul K. (2017). Standard comparison of local mental health care systems in eight European countries. Epidemiol. Psychiatr. Sci..

